# Molecular Insights from Differential Proteomic Profiling of Premalignant Cervical Lesions and Cervical Cancer

**DOI:** 10.3390/pathogens15080793

**Published:** 2026-07-26

**Authors:** Diana Laura Gonzalez-Tolentino, Olga Lilia Garibay-Cerdenares, Sergio Encarnación-Guevara, Ángel Gabriel Martínez-Batallar, Ramiro Alonso-Bastida, Jeovanis Gil, Jorge Organista-Nava, Luz del Carmen Alarcón-Romero, Marco Antonio Leyva-Vázquez, Berenice Illades-Aguiar

**Affiliations:** 1 Laboratorio de Biomedicina Molecular, Facultad de Ciencias Químico Biológicas, Universidad Autónoma de Guerrero, Chilpancingo 39090, Guerrero, Mexico; 11082612@uagro.mx (D.L.G.-T.); olgaribayce@secihti.mx (O.L.G.-C.); jorgeorganista@uagro.mx (J.O.-N.); 2 Secretaria de Ciencia, Humanidades, Tecnología e Innovación, Investigadores por México, Laboratorio de Biomedicina Molecular, Facultad de Ciencias Químico Biológicas, Universidad Autónoma de Guerrero, Chilpancingo 39090, Guerrero, Mexico; 3 Centro de Ciencias Genómicas, Universidad Nacional Autónoma de México, Cuernavaca 62210, Morelos, Mexico; encarnac@ccg.unam.mx (S.E.-G.); angelmb@ccg.unam.mx (Á.G.M.-B.); rbastida@ccg.unam.mx (R.A.-B.); 4 Section for Clinical Chemistry, Department of Translational Medicine, Lund University, 22242 Lund, Sweden; jeovanis.gil_valdes@med.lu.se; 5 Laboratorio de Investigación en Citopatología e Histoquímica, Facultad de Ciencias Químico Biológicas, Universidad Autónoma de Guerrero, Chilpancingo 39090, Guerrero, Mexico; lcalarcon@uagro.mx

**Keywords:** LSIL, SCC, HPV16, proteomic, S100A10 and TYMP

## Abstract

Cervical cancer (CC) affects women worldwide, and more than 95% of cases are caused by persistent infection with high-risk human papillomavirus (HR-HPV), such as type 16, which promotes the progression of precancerous lesions to cancer. This study aimed to identify differentially expressed proteins (DEPs) in biopsies from patients with HPV16+ low-grade squamous intraepithelial lesions (LSILs) and from patients with HPV16+ squamous cell carcinoma (SCC) compared with those from HPV-negative normal cervical tissue (NCT HPV−) controls. The samples were analyzed by high-performance liquid chromatography–tandem mass spectrometry (HPLC-MS/MS) using a data-independent acquisition (DIA) approach. Data processing and differential protein expression analysis were performed with the DIA-NN software (Data-Independent Acquisition Neural Networks), followed by bioinformatics analyses, including Venn diagrams, pathway enrichment, functional interactome, The Cancer Genome Atlas (TCGA)-SCC data integration, and Western blot detection. In total, 1607 DEPs associated with cell adhesion and extracellular matrix proteins were identified in LSILs, whereas 1516 DEPs associated with catalytic and transport activities were identified in SCC; the proteins overexpressed in LSILs (332) were enriched in processes such as metabolism, immune response activation, and stress and cell death responses. In contrast, proteins overexpressed in SCC (205) were associated with the cell cycle, DNA damage, drug metabolism, proteasome degradation, methylation, and immune response. Interaction analyses highlighted proteins related to early proteins 1,5,6 and 7 (E1, E5, E6, and E7). In terms of the two DEPs, S100 calcium binding protein A10 (S100A10/p11) and thymidine phosphorylase (TYMP) were detected in patients with LSIL, HSIL, and SCC at the protein level, consistent with their higher transcript levels in public datasets. Given the small, exploratory cohort, these findings are hypothesis-generating, and validation in a larger, balanced, independent cohort is required. In conclusion, this study identified DEPs associated with the progression of premalignant lesions to SCC that may represent candidate biomarkers and therapeutic targets warranting further investigation.

## 1. Introduction

Cervical cancer (CC) is the fourth most common cancer in women, with an incidence of 600,000 cases and 348,189 deaths worldwide [1]. Approximately 95% of cervical cancer cases are associated with high-risk human papillomavirus (HR-HPV) infection [2]. This is due primarily to oncogenic HPV16, which is detected in 65–72% of squamous cell carcinoma (SCC) cases [3]. The progression of precancerous lesions to more advanced stages of the disease is driven by host risk factors (age, multiparity, and number of sexual partners, among others) and by viral characteristics (viral genotype, coinfections, viral load, and integration) [4]. The overexpression and direct or indirect interactions of HR-HPV E5, E6, and E7 oncoproteins with host proteins affect molecular mechanisms such as cell proliferation, metabolism, apoptosis, differentiation, and genomic instability, processes that are considered hallmarks of cervical cancer progression [5]. In omics studies, proteomics has enabled the identification of biomarkers, the elucidation of molecular mechanisms associated with disease progression, and the discovery of new therapeutic targets [6]. Proteomics studies have been performed using various biological conditions, such as biopsies [7], cervicovaginal fluid [8], and body fluids such as serum [9], and using various clinical characteristics, histological classifications, HPV genotypes, and disease stages. These studies have allowed the identification of a wide variety of differentially expressed proteins that could be validated; however, the significant clinical variability and different data analysis strategies have complicated the validation process because of interindividual variability among patients. Several proteomic studies have been conducted in cell lines [10] to examine the molecular mechanisms underlying proliferation [11], metastasis [6], signaling pathways [12], and drug resistance [13]. However, through various omics studies on cervical cancer, molecular signatures associated with SCC progression have been identified, such as the presence of mutations in phosphatidylinositol-4,5-bisphosphate 3-kinase catalytic subunit alpha (PIK3CA), which promote glycolysis and cell proliferation, in addition to mutations in genes such as MYC proto-oncogene (MYC), erb-b2 receptor tyrosine kinase 2 (ERBB2), GLI family zinc finger 2 (GLI2)*,* NCK interacting kinase (TNIK), nuclear receptor subfamily 4 group A member 2 (NR4A2), prospero homeobox 1 (PROX1), argonaute RISC catalytic component 2 (AGO2), TOG array regulator of axonemal microtubules 1 (FAM179B) and Serpin family B member 4 (SERPINB4) that increase their expression as a function of HPV integration in SCC [14]. Molecular signatures associated with therapeutic resistance have been identified in SCC, in which overexpression of mucin 4 and 12, cell surface associated (MUC12, MUC4), microtubule actin crosslinking factor 1 (MACF1), obscurin, cytoskeletal calmodulin and titin-interacting RhoGEF (OBSCN), Fibrocystin-L/Polycystic kidney and hepatic disease 1-like protein 1 (PKHD1L1), plectin (PLEC), and LDL receptor related protein 1B (LRP1B) was associated with radiotherapy resistance [15].

Through integrated omics studies using tools such as genomics, transcriptomics, and proteomics (phosphoproteomics and acetylproteomics), a new molecular stratification method based on proteomics was proposed, correlating prognostic data, genetic alterations, immune infiltration, and some post-translational modifications, revealing that post-translational modifications (PTMs) such as the acetylation of FOS-like antigen 2/acetylation at lysine 222 (FOSL2-K222) by E1A binding protein p300 (EP300) were associated with proliferation in CC and that the overexpression of protein kinase C beta (PRKCB) could be helpful as a marker of the response to chemotherapy [16]. Proteomics has also enabled the identification of proteins with a relevant role as biomarkers in the progression of early lesions to cancer, as is the case for minichromosome maintenance 10 replication initiation factor (MCM10) in CC [17].

However, these studies have primarily been conducted in Asian and European populations, whereas in Latin American countries, the number of proteomic studies is minimal. A survey of CC cell lines (HeLa, CaLo, SiHa, Caski, ViBo, and C-33A) using two-dimensional electrophoresis and matrix-assisted laser desorption/ionization time-of-flight mass spectrometry (MALDI–TOF–MS) revealed 14-3-3 protein zeta/delta (14-3-3ζ) as a key player in malignant transformation [18]. In another study in which biopsies from patients with SCC were compared with those from normal cervical cells by two-dimensional difference gel electrophoresis (2D-DIGE) and MALDI–TOF–MS, osteoglycin (OGN), actin alpha 2, smooth muscle (ACTA2), lumican (LUM), peroxiredoxin 1 (PRDX1), stratifin (SFN), enolase 1 (ENO1), and keratin 5 (KRT5) were identified as differentially expressed proteins and potential therapeutic targets [7].

In this work, by means of proteomics (HPLC-MS/MS), data-independent acquisition and bioinformatics analysis, DEPs were identified in LSIL HPV16+ and SCC HPV16+ cervical tissue compared with NCT HPV− related to processes associated with the progression of premalignant lesions to cancer, revealing their relationships with viral oncoproteins E5, E6, and E7 and their probable relationships with chemotherapeutic drugs used for the treatment of CC that could be associated with chemoresistance.

## 2. Materials and Methods

### 2.1. Patients with LSILs, SCC HPV16+, and NCT HPV- and Ethical Statements

Biopsies were from the biobank of the Laboratory of Molecular Biomedicine of the Faculty of Chemical–Biological Sciences of the Autonomous University of Guerrero (UAGro) belonging to the National Laboratory of Biobanks (LANBIOBAN) [19]. The biopsies were collected with informed consent and in accordance with the protocol approved by the Subdirección de Enseñanza del Instituto Estatal de Cancerología Dr. Arturo Beltrán Ortega de Acapulco, Guerrero (DGIECAN/CCHGC/CEI/PRO-039-2024: approval date: 25 April 2024). The biopsies came from patients who were captured for the first time (newly diagnosed) and who did not receive any chemotherapy treatment.

Patient samples were included based on the following criteria: (a) samples from patients with histopathologically confirmed diagnoses of low-grade intraepithelial lesion (LSIL) and squamous cell cervical carcinoma (SCC), (b) with HPV16 infection determined by the Innogenetics line probe assay (HPV genotyping) (INNO-LIPA) (HPV16+) and (c) HPV-negative normal cervical tissue (NCT HPV-).

For the proteomic discovery phase, 20 biopsies were processed: NCT HPV− (n = 10), LSIL HPV16+ (n = 3), and SCC HPV16+ (n = 7), one biopsy per patient. After protein extraction, all 20 samples reached the required quantity (≥50 µg) for reduction, alkylation, and digestion; during HPLC-MS/MS, one specimen (an SCC sample) failed to achieve adequate chromatographic separation and was excluded. Consequently, 19 final specimens (NCT HPV− n = 10; LSIL HPV16+ n = 3; SCC HPV16+ n = 6) met all quality control criteria and were included in the final proteomic analysis. For Western blot, 8 of 19 of these specimens had sufficient protein (≥60 µg); 4 additional biopsies were incorporated (1 LSIL, 2 HSIL, 1 SCC), giving a total of 12 specimens: LSIL (3), HSIL (2), SCC (3) and NCT HPV-(4). The clinical data of specimens used for HPLC-MS/MS and WB are provided in Supplementary Table S1.

### 2.2. Protein Extraction from TRIzol^®^

Protein extraction was performed from the organic phase obtained after cell lysis using TRIzol^®^ (Thermo Scientific, Waltham, MA, USA) according to the manufacturer’s recommendations. For each milliliter of TRIzol^®^ used in the lysis, 0.3 mL of absolute ethanol was added; the mixture was incubated and centrifuged at 2000× *g* for 5 min at 4 °C to precipitate deoxyribonucleic acid (DNA). To the resulting supernatant, 1.5 mL of cold isopropanol was added to each milliliter of TRIzol^®^ used. The mixture was subsequently centrifuged at 12,000× *g* for 10 min at 4 °C. The protein pellet obtained was subsequently washed with 2 mL of 0.3 M guanidine chloride (Sigma-Aldrich 50-01-1; St. Louis, MO, USA) in 95% ethanol. The samples were incubated and centrifuged at 7500× *g* for 5 min at 4 °C. The supernatant was removed, and the pellet was dried for 5–10 min. The proteins were resuspended in 1% Tris-SDS buffer and stored at −80 °C until further analysis.

### 2.3. Protein Quantification

Total extracts were quantified using the Pierce 660 nm method (Thermo Fisher Scientific, Rockford, IL, USA) in a microplate according to the manufacturer’s protocol. Briefly, 10 µL of each bovine serum albumin standard (125, 250, 500, 750, 1000, 1500, and 2000 µg/mL) and 10 µL of the test samples and the blank (SDS-Tris 1% lysis buffer) were added in triplicate, after which 150 µL of the protein reagent was added to each well, and the reading was performed on a Multiskan FC plate reader (Thermo Fisher Scientific, Waltham, MA, USA) after 5 min of shaking. The absorbance was measured at 660 nm. The protein concentration was determined prior to reduction, alkylation, and enzymatic digestion. In addition, the peptide concentration was measured using a NanoDrop spectrophotometer 2000c Spectrophotometer(Thermoscientific, Whaltham, MA, USA) before liquid chromatography–tandem mass spectrometry (LC-MS/MS) analysis.

### 2.4. Digestion, Reduction, and Alkylation

Fifty micrograms of the precipitated protein extract were washed three times with 90% ethanol and resuspended in 50 µL of Tris (pH 8.8). Once dissolved, reduction was carried out with dithiothreitol (DTT) to a final concentration of 100 mM, and the mixture was incubated at 56 °C for 30 min, followed by alkylation with iodoacetamide to a final concentration of 200 mM and incubation in the dark for 30 min. The modified proteins were solubilized in 50 mM ammonium bicarbonate and 0.5% deoxycholate, after which 1 µg of modified porcine trypsin (purity grade) was added. Digestion was carried out at 37 °C for 16 h. Detergent extraction was performed with ethyl acetate and trifluoroacetic acid at a final concentration of 0.5%. The organic phase was centrifuged and removed. Finally, the peptides were dried in a SpeedVac and stored at −80 °C until analysis.

### 2.5. Analysis by LC-MS/MS

#### 2.5.1. Identification/Quantification of Peptides/Proteins

As previously described, [20] 1 µg of the peptide mixtures was analyzed using a liquid chromatography-mass spectrometry system composed of a Dionex Ultimate 3000 RSLCnano ultra-performance liquid chromatography (UPLC) coupled to a Q Exactive HF-X mass spectrometer. The mass spectrometer was operated in positive mode, and the data were acquired using an in-house-developed variable-window data-independent acquisition method. Peptides were loaded and desalted on an Acclaim PepMap100 C18 trap column (3 µm, 100 Å, 75 µm i.d. × 2 cm, nanoViper) with aqueous 0.1% trifluoroacetic acid (TFA) at a flow rate of 5 µL/min for 6 min. Peptide separation was performed in-line, connecting the trap column to an EASY-spray RSLC C18 analytical column (2 µm, 100 Å, 75 µm i.d. × 50 cm) at a flow rate of 300 nL/min. The trap and analytical columns were set to 35 °C and 60 °C, respectively. Peptides were separated using a nonlinear gradient over 60 min, starting from 2 to 27% buffer B in 55 min, then from 27 to 35% and from 35 to 55% over 2 min each, and finally reaching 90% buffer B in 1 min. The acquisition method consisted of a complete acquisition cycle comprising 54 variable-width mass spectrometry stage 1/stage 2 (MS2) scans and 3 (MS1) scans over the mass range of 385–1460 m/z. The MS2 window widths were determined from the empirical distribution of signals from data-dependent acquisition (DDA) runs with similar samples, with a 1 Da overlap between adjacent windows. The parameters for MS1 were as follows: resolution of 120,000 (@ 200 m/z), automatic gain control (AGC) target of 3 × 10^6^, and maximum injection time of 50 ms. For MS2, the resolution was set to 30,000, with an AGC target of 1 × 10^6^, automatic maximum injection time, and normalized collision energy (NCE) of 28.

#### 2.5.2. Bioinformatic Analysis for Peptide and Protein Identification

Raw DIA files were processed with DIA-NN v1.8.1 [21] for precursor search and fragment identification using two-dimensional peak positioning (retention time and precursor *m*/*z*), with spectral library prediction via neural networks. Key search parameters included: protein inference = “Genes”, quantification = “Robust LC (High precision)”, cross-run normalization = “RT-dependent”, with MBR (Match Between Runs), isotopologue handling, and heuristic protein inference enabled. Searches were performed against the UniProt human proteome (UP000005640, released 27 July 2024), using trypsin digestion, fixed carbamidomethylation of cysteines, and variable methionine oxidation. Data harmonization was performed with MS-DAP v1.1 [22], retaining only samples with ≥3 detected peptides. Normalization combined variance stabilizing normalization (VSN) [23] and the “modebetween_protein” method. Differential expression was assessed across three contrasts—NCT HPV− vs. LSIL, NCT HPV− vs. SCC, and LSIL vs. SCC—using msempire [24], with contrast-specific peptide filtering applied to maximize the number of peptides available per comparison. The NCT HPV− vs. LSIL contrast included 26,398 peptides across 4395 proteins; NCT HPV− vs. SCC included 25,911 peptides across 4041 proteins; and LSIL vs. SCC included 27,461 peptides across 4420 proteins. Differentially expressed proteins (DEPs) were defined by an adjusted *p*-value (FDR) < 0.05 and a bootstrap-estimated |log_2_ fold change| threshold specific to each contrast (≥0.818 for NCT HPV− vs. LSIL, ≥0.863 for NCT HPV− vs. SCC, and ≥1.068 for LSIL vs. SCC). Principal component analysis (PCA) was computed on peptides consistently detected within each group to assess global sample variation while minimizing the influence of sporadically detected or low-abundance features.

These tools and thresholds were selected to control data quality given the small cohort. DIA-NN was used for its neural network–based processing and interference correction, which provide deep, reproducible proteome coverage in label-free DIA data; the retention of samples with ≥3 detected peptides in MS-DAP excludes poorly sampled runs; VSN combined with the “modebetween_protein” method stabilizes the mean–variance relationship across the intensity range and corrects systematic between-sample shifts, which is robust when the number of replicates is small; and msempire models peptide-level noise distributions, providing sensitive and well-calibrated detection of DEPs in small cohorts. The significance and effect-size thresholds (adjusted *p*-value (FDR) < 0.05 and the bootstrap-estimated, contrast-specific |log_2_ fold change|) were set to control the false discovery rate while accounting for the limited sample size.

For the comparative analyses among conditions (NCT HPV− vs. LSIL, NCT HPV− vs. SCC, and LSIL vs. SCC) used to construct the Venn diagrams and the functional enrichment interactomes, a more stringent, uniform fold change filter was subsequently applied to the DEPs, retaining only proteins with log_2_FC ≥ +1 (overexpressed) or log_2_FC ≤ −1 (underexpressed).

### 2.6. Bioinformatic Analysis

For the comparative analyses (Venn diagrams and functional enrichment interactomes) and in Supplementary Figures S1 and S2, a more stringent, uniform fold change filter was subsequently applied to the DEPs, retaining only proteins with log_2_FC ≥ +1 (overexpressed) or log_2_FC ≤ −1 (underexpressed): LSIL 1314 DEPs, SCC 1307 DEPs, and LSIL vs. SCC 217 DEPs. UniProt IDs were extracted from the list of differentially expressed proteins (www.uniprot.org). *Homo sapiens* protein–protein interactions were retrieved using stringApp (v2.2.0) within Cytoscape (minimum confidence score 0.4). Because viral proteins are not part of the human STRING network, the HPV16 proteins (E1, E5, E6, E7; NCBI taxonomy ID 333760) were obtained from viruses. STRING and linked to their human host targets, and the resulting networks were visualized in Cytoscape (v3.10.2). Functional enrichment of the network nodes was computed with the STRING Enrichment module (stringApp) against the Gene Ontology “Biological Process” annotation, using the DEPs as input and an adjusted *p*-value (FDR) < 0.05 as the significance threshold. The drug–protein interactome (cisplatin, paclitaxel) was built from STITCH (v5.0) via stringApp. An analysis of overlapping common and unique proteins was performed on the BioVenn server (https://www.biovenn.nl/). Supplementary Table S3 includes data on the functional enrichment of DEPs and the predicted processes associated with under- and overexpression, as well as shared proteins.

### 2.7. TCGA Dataset

The Cervical Cancer Genome Atlas Research Network (TCGA-CESC) was used to visualize the clinical data. The cBioPortal resource (https://www.cbioportal.org/) provided access to data from the “Cervical Squamous Cell Carcinoma (TCGA, PanCancer Atlas)” database (access date: 16 November 2025), including information from 297 patients and 297 samples, and we downloaded clinical data from all TCGA samples. Additionally, the University of Alabama Cancer Database (UALCAN) (https://ualcan.path.uab.edu/, access date: 15 December 2025) was used to compare the expression of S100A10 and TYMP in squamous cell carcinoma versus paratumoral cervical tissue from the CESC-TCGA dataset.

### 2.8. Determination of Expressed Proteins: Western Blot

From whole-cell lysates (60 µg), S100A10 (~11 kDa) was resolved on 18% SDS-PAGE gels, whereas TYMP (~55 kDa) and GAPDH (~37 kDa) were resolved on 10% SDS-PAGE gels and transferred to nitrocellulose membranes. To block nonspecific binding, the membranes were incubated in 5% (*w*/*v*) nonfat dried milk in Tris-buffered saline (TBS)/T (0.05% Tween 20 in TBS) for 2 h at room temperature (RT). After blocking, the membranes were incubated overnight at 4 °C with a primary antibody against S100A10 (clone 4E7E10; Cat. No. sc-81153; Santa Cruz Biotechnology, Dallas, TX, USA; MW: ~11 kDa, dilution 1:250), TYMP (PD-ECGF) (clone PGF-44C; Cat. No. sc-47702; Santa Cruz Biotechnology, Dallas, TX, USA; MW: ~55kDa; dilution 1:250), and glyceraldehyde-3-phosphate dehydrogenase (GAPDH) (clone H-12; Cat. No. sc-166574; Santa Cruz Biotechnology, Dallas, TX, USA; MW: ~37kDa; dilution 1:1000). After incubation with the primary antibody, the membranes were washed and incubated with a horseradish peroxidase (HRP)-conjugated goat anti-mouse secondary antibody (IgG-HRP; Cat. No. sc-2005; Santa Cruz Biotechnology, Dallas, TX, USA; dilution 1:2500) for 2 h at RT. The immunoreaction was revealed with a chemiluminescent substrate (Supersignal West Femto Luminol, Thermo Scientific, Waltham, MA, USA, Cat. No. 1856189) on radiographic films (HyBlot CL, Cat. No. 1141J52, Thomas Scientific, Waltham, MA, USA, LLC) using AGFA DENTUS-1000 developer/fixer solutions (REF 369US, REF 369VU, Kulzer, Hanau, Germany). Densitometric analysis of the images was performed in ImageJ 1.54p and analyzed with GraphPad Prism 8.0.1.

### 2.9. Statistical Analysis

Statistical analyses were performed in GraphPad Prism 8.0.1. Group differences in the proteomic relative abundances and in the Western blot densitometry were assessed by the Kruskal–Wallis test followed by Dunn’s multiple-comparisons test. Data are presented as medians with interquartile ranges, and a two-sided *p* < 0.05 was considered statistically significant.

## 3. Results

### 3.1. Proteomic Analysis in Patients with LSIL and SCC Who Were HPV16+ Compared with Those with NCT HPV−

**Figure 1:**
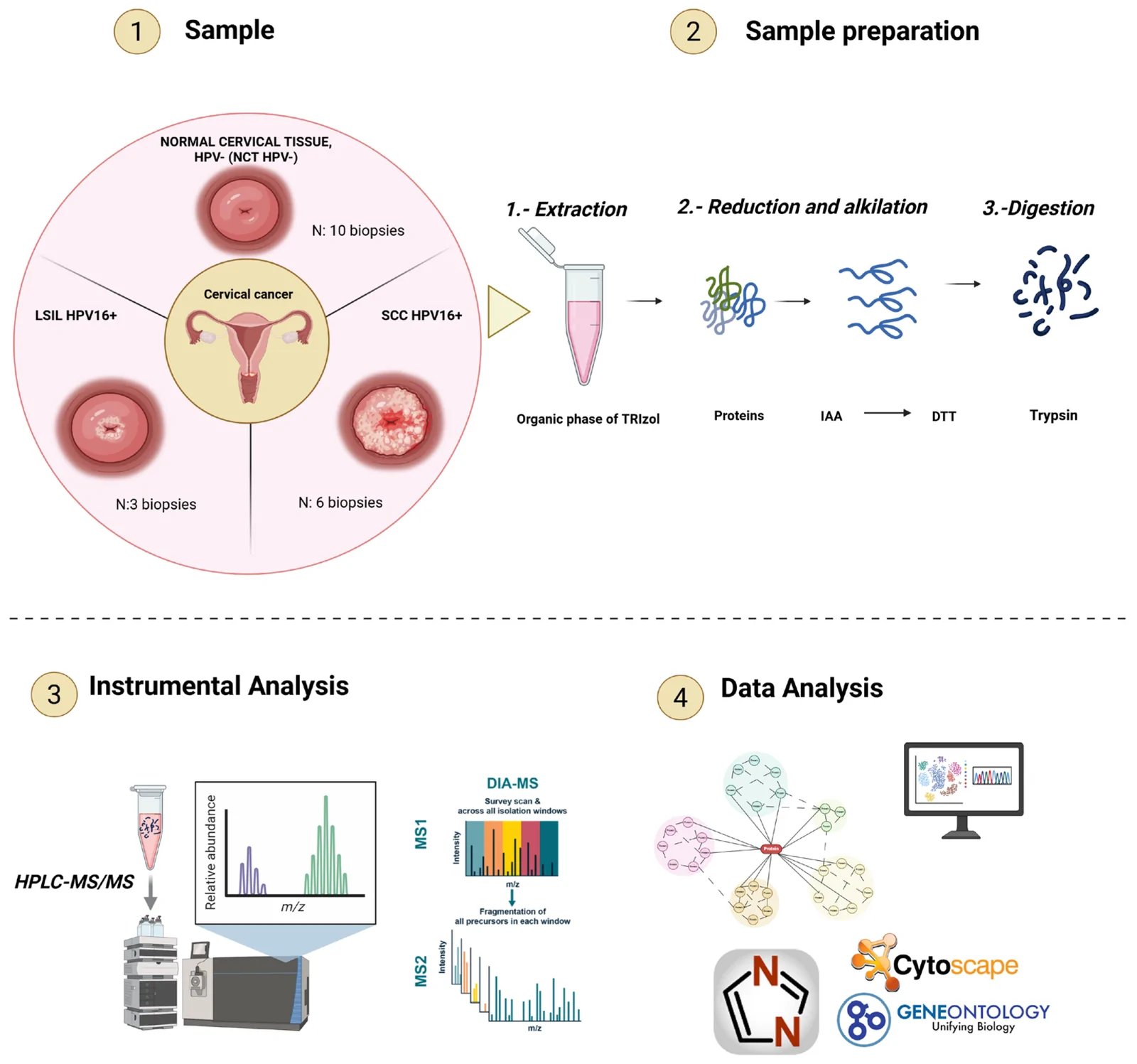
Overview of the experimental strategy for proteomic analysis. (1) Biopsies from patients classified as NCT HPV−, LSIL HPV16+, and SCC HPV16+ were obtained. (2) Extraction, quantification, and digestion of proteins were performed to obtain peptides. (3) HPLC-MS/MS with DIA-MS. (4) Identification and bioinformatic integration of differentially expressed proteins and analysis of interaction networks.

For comparative proteomic analysis, biopsies classified as normal cervical tissue (NCT HPV−), low-grade intraepithelial lesions (LSIL HPV16+), and squamous cell carcinoma (SCC HPV16+) were included. From these samples, extraction, quantification, reduction, alkylation, and protein digestion were performed to obtain peptides, which were analyzed by HPLC-MS/MS. The results were evaluated using DIA-MS. The results obtained were subjected to bioinformatics analysis to identify DEPs, and functional enrichment analyses were performed in addition to generating protein-protein interaction networks, considering host proteins, viral proteins, protein-drug interactions, and finally, the chemotherapeutics used for the treatment of CC, which were based on cisplatin and paclitaxel (Figure 1).

### 3.2. Differential Protein Expression (DEP) in LSILs and SCC HPV16+ Cells

**Figure 2:**
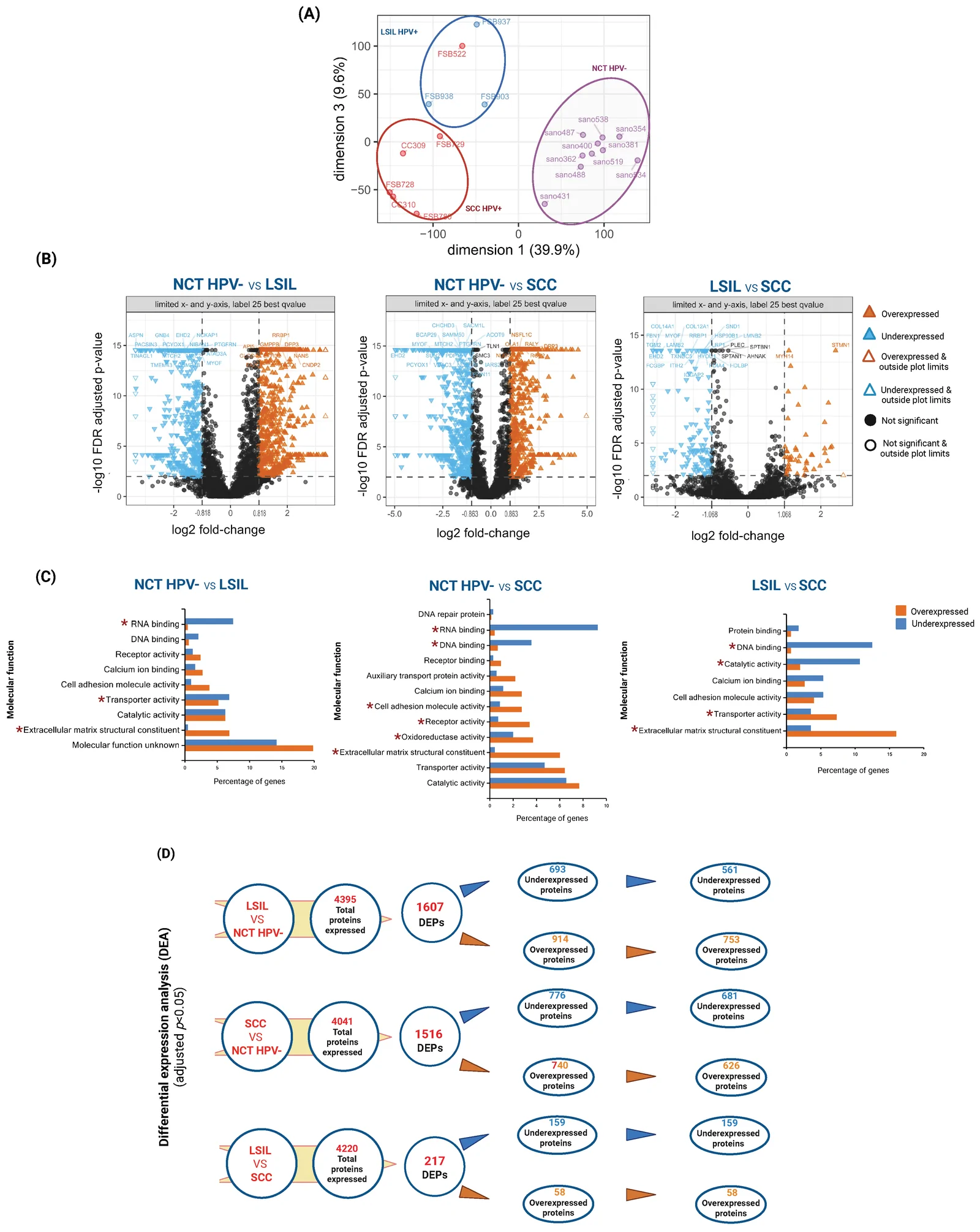
Differential analysis of the NCT HPV−, LSIL HPV16+, and SCC HPV16+ proteomes. (**A**) Principal component analysis (PCA) of the NCT HPV−, LSIL HPV16+, and SCC HPV16+ study groups. (**B**) Volcano plots showing overexpressed (orange) and underexpressed (blue) proteins in the NCT HPV− vs. LSIL HPV16+; NCT HPV− vs. SCC HPV16+; and LSIL HPV16+ vs. SCC HPV16+ groups (adjusted *p* < 0.05: |log2 FC| ≥ 0.818 for NCT HPV− vs. LSILs, |log2 FC| ≥ 0.863 for NCT HPV− vs. SCC and, |log2 FC|≥ 1.068 for LSILs vs. SCC). Dashed lines indicate the significance threshold (adjusted *p*-value (FDR) = 0.05) and the contrast-specific |log2 fold change| cutoffs (≥0.818 for NCT HPV− vs. LSIL; ≥0.863 for NCT HPV− vs. SCC; ≥1.068 for LSIL vs. SCC). Orange, overexpressed; blue, underexpressed; black, non-significant (**C**) GO-based functional enrichment using the Funrich tool showing the most-enriched terms per comparison; blue bars correspond to underexpressed proteins and orange bars to overexpressed proteins, consistent with the color code in panel (**B**). (**D**) Schematic of the total number of DEPs in comparisons between NCT HPV−, LSIL HPV16+, and SCC HPV16+. From the DEPs, a more stringent subset was selected for the bioinformatic analyses, selecting those with an adjusted *p*-value (FDR) < 0.05 and a log_2_FC ≥ +1 (overexpressed) or ≤−1 (underexpressed). * Significant processes.

To identify the differential expression profile between LSILs and SCC, in comparison with NCT HPV−, principal component analysis (PCA) was performed, which revealed that the two components (dimensions one and three) explained more than 39.9% of the variation among the samples analyzed, which allowed the groups to be separated according to their proteomic profiles and revealed three comparative conditions, namely, NCT HPV−, LSILs, and SCC (Figure 2A).

DEPs revealed three groups: LSIL HPV16+ and SCC HPV16+ (both in comparison with NCT HPV−), and a third analysis that compared SCC HPV16+ (with respect to LSIL-HPV16+) to identify unique and shared proteins between low-grade lesions and cancer. Under these criteria, for LSIL HPV16+, 4395 proteins were identified, of which 1607 were considered DEPs (adjusted *p* < 0.05, |log2 FC| ≥ 0.818 for NCT HPV− vs. LSILs), and of which 693 were underexpressed, while 914 were overexpressed. In SCC, 4041 HPV16+ proteins were identified, of which 1516 were considered DEPs (adjusted *p* < 0.05, |log2 FC| ≥ 0.863 for NCT HPV− vs. SCC); of these, 776 were underexpressed, and 740 were overexpressed. Finally, in the comparison between SCC and LSIL HPV16+, 4420 proteins, of which 217 DEPs (adjusted *p* < 0.05, |log2 FC| ≥ 1.068 for LSILs vs. SCC), were identified, and of these 159 proteins were underexpressed, while 58 were overexpressed (Figure 2B). When the overall functional processes enriched in LSIL HPV16+ cells were analyzed, most of the underexpressed functions were related to ribonucleic acid (RNA) binding, transport, and catalytic activity. In contrast, the processes enriched in the overexpression group included cell adhesion, receptors, and extracellular matrix components, among others. On the other hand, in HPV16+ SCC cells, downregulation was associated with RNA and DNA binding, catalytic activity, and transport activity. In contrast, overexpression was associated with processes such as cell adhesion, transporter and catalytic activity, extracellular matrix components, and oxidoreductase activity. However, when SCC (with respect to LSILs) was compared, the processes associated with underexpression were DNA binding, catalytic activity, calcium ion binding, and cell adhesion molecules. In contrast, overexpressed proteins were related to extracellular matrix components, catalytic and transporter activity (Figure 2C). 

For the comparative analyses among conditions of LSIL, SCC, and SCC in comparison with LSIL, Venn diagrams and functional enrichment interactomes, a more stringent, uniform fold change filter was subsequently applied to the DEPs, retaining only proteins with an adjusted *p*-value (FDR) < 0.05 and log_2_FC ≥ +1 (overexpressed) or log_2_FC ≤ −1 (underexpressed). This two-fold cutoff focuses the biological process interpretation on the most strongly regulated proteins; the resulting counts are: for LSIL, 561 subexpressed and 753 overexpressed; for SCC, 681 subexpressed, and 626 overexpressed; and, finally, for LSILs vs. SCC, 159 underexpressed and 58 overexpressed (Figure 2D). 

Overall, LSIL and SCC display distinct DEP repertoires, with cell adhesion, extracellular matrix remodeling, and RNA binding predominating in LSIL and catalytic, transport, and RNA binding predominating in SCC.

### 3.3. Cellular Processes Associated with Under- and Overexpression in LSILs and SCC HPV16+ Cells

**Figure 3:**
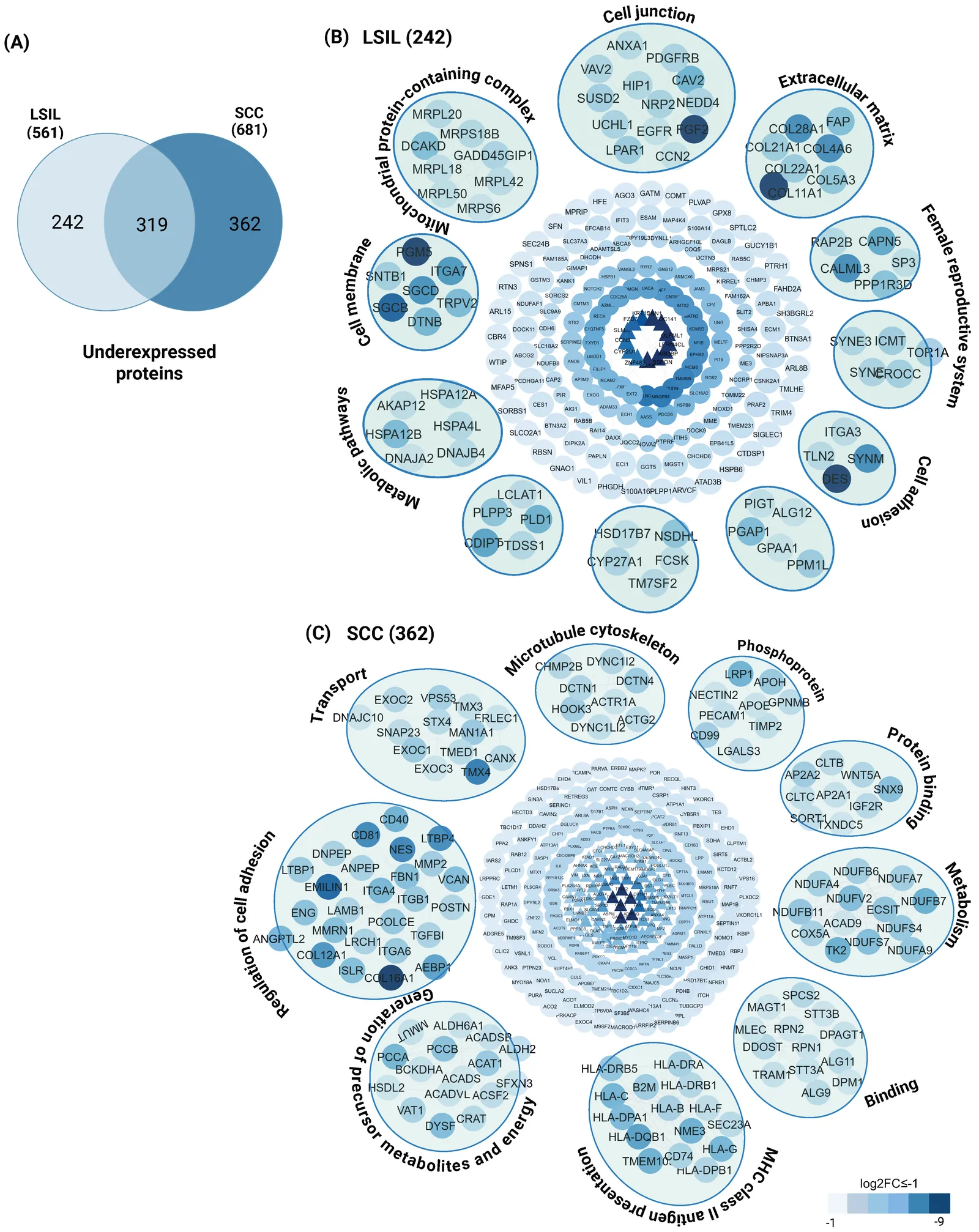
Bioinformatic analysis of LSIL HPV16+ and SCC HPV16+ underexpressed proteins. (**A**) Venn diagram of LSIL HPV16+ and SCC HPV16+ underexpressed proteins. (**B**,**C**) Interactome of the 242 exclusively underexpressed proteins in LSIL HPV16+ and 362 exclusively underexpressed proteins in SCC-HPV16+ associated with biological processes. Blue color intensity is associated with underexpression. Circles and triangles correspond to DEPs (log_2_FC ≤ −1, underexpressed). The visual encoding of the nodes (node color with underexpression) and node size with biological process enrichment.

Once the DEPs (adjusted *p* < 0.05, |log2 FC| (≥0.818, LSIL and ≥0.863, SCC)) in each comparative group were obtained, significant DEPs (log_2_FC ≤ −1, underexpressed, and log_2_FC ≥ +1, overexpressed) were selected for bioinformatic analysis. Under these criteria, LSIL HPV16+ was associated with 561 underexpressed DEPs, whereas SCC HPV16+ was associated with 681 underexpressed DEPs. The results showed 319 common proteins; 242 were exclusive to LSIL, whereas 362 were exclusive to SCC (Figure 3A). The pathway enrichment analysis using a functional interactome of proteins specific to each group revealed that processes downregulated only in LSILs were associated with reproductive health: calmodulin-like protein 3 (CALML3), calpain 6 (CAPN6), protein phosphatase 1 regulatory subunit 3D (PPP1R3D), cell membrane proteins: phosphoglucomutase-like protein 5 (PGM5), beta-sarcoglycan (SGCB), delta-sarcoglycan (SGCD) and integrin subunit alpha 7 (ITGA7), cell junctions: fibroblast growth factor 2 (FGF2) and caveolin-2 (CAV2), and metabolic pathways phospholipase D1 (PLD1), CDP-diacylglycerol–inositol 3-phosphatidyltransferase (CDIPT) and phospholipid phosphatase 3 (PLPP3) (Figure 3B). On the other hand, the processes in which expression exclusively decreased in SCC included the adaptive immune system: HLA class II histocompatibility antigen, DQ beta chain (HLA-DQB1), transmembrane protein 109 (TMEM109) and nucleoside diphosphate kinase 3 (NME3), transport: thioredoxin-related transmembrane protein 4 (TMX4), precursor metabolite generation and energy pathways: propionyl-CoA carboxylase alpha chain, mitochondrial (PCCA-B), acetyl-CoA acetyltransferase, mitochondrial (ACAT1) and dysferlin (DYSF), and regulation of cell adhesion: collagen alpha-1 (XVI) chain (COL16A1), EMILIN-1/elastin microfibril interfacer 1 (EMILIN1) and nestin (NES), among others (Figure 3C).

Among the 319 DEPs shared between HPV16+ LSIL and HPV16+ SCC and associated with underexpression, the enriched processes were cell adhesion: integrin subunit beta 5 (ITGB5), laminin subunit beta 2 (LAMB2), collagen alpha-2 (VI) chain (COL6A2) and tenascin-X (TNXB), regulation of migration: caveolin-1 (CAV1) and decorin (DCN), and metabolic pathways: annexin A6 (ANXA6), transgelin (TAGLN) and guanine nucleotide-binding protein subunit beta-1,2 and 4 (GNB1/2/4). This shared downregulated set points to a common loss of structural and adhesion-related functions in both lesion stages (Supplementary Figure S1).

**Figure 4:**
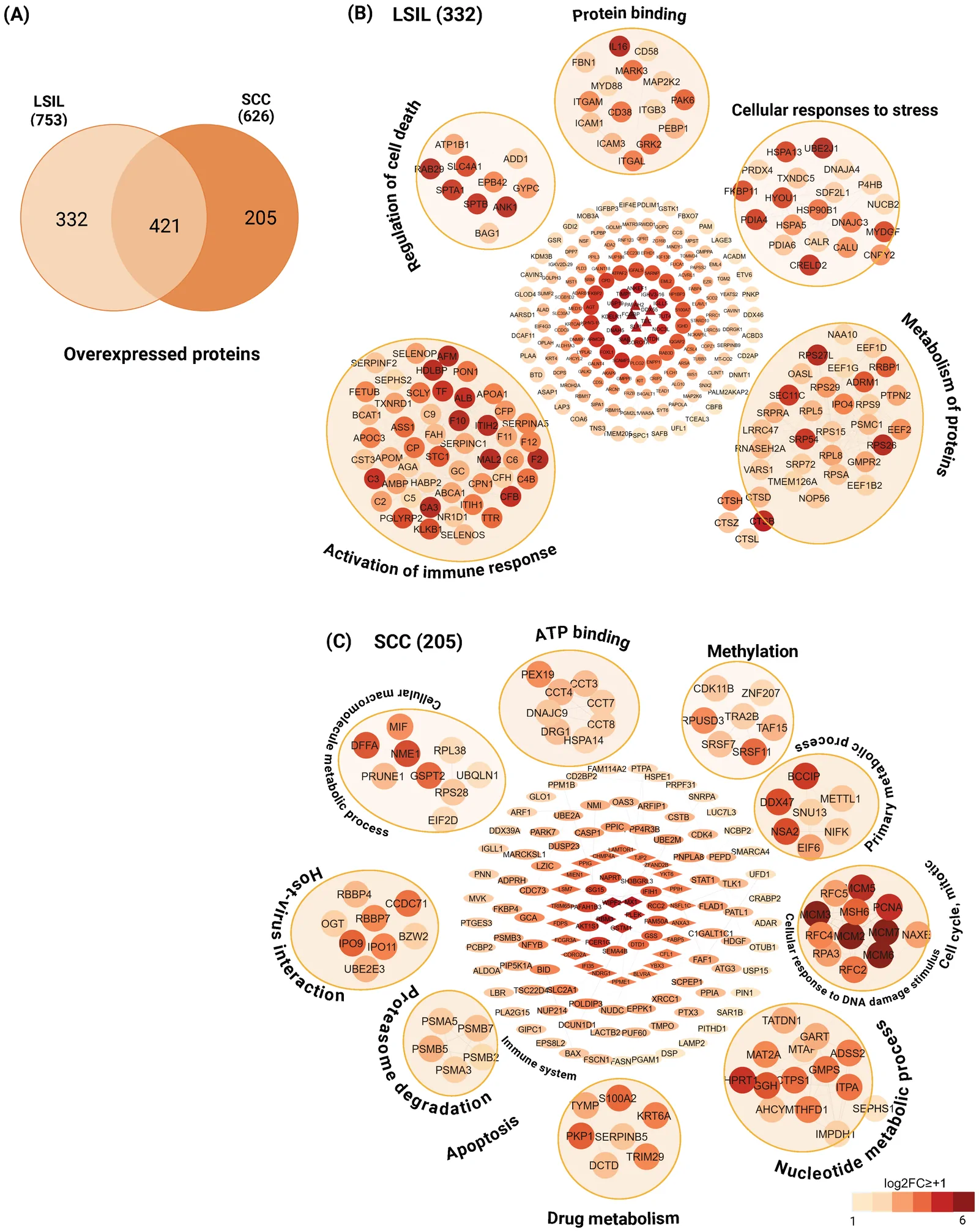
Bioinformatic analysis of proteins overexpressed in LSIL HPV16+ and SCC HPV16+ cells. (**A**) Venn diagram of proteins overexpressed in LSIL HPV16+ and SCC HPV16+ cells. (**B**,**C**) Interactome of the 332 overexpressed proteins unique to LSIL HPV16+ and 205 proteins unique to SCC-HPV16+ associated with biological processes. Orange color intensity is associated with overexpressed proteins. Circles and triangles correspond to DEPs (log_2_FC ≥ +1, overexpressed). The visual encoding of the nodes (node color with underexpression) and node size with biological process enrichment.

However, when the DEPs associated with overexpression were analyzed, 753 were detected in LSIL HPV16+, and 626 were detected in SCC HPV16+. In the Venn diagrams, 332 exclusive proteins were detected in LSILs, and 205 were detected in SCC (Figure 4A). Functional enrichment analysis revealed that the exclusive proteins in LSILs were enriched in processes related to protein metabolism: 40S ribosomal protein S26/small ribosomal subunit protein eS26 (RPS26), 40S ribosomal protein S27-like (RPS27L), signal peptidase complex catalytic subunit SEC11C (SEC11C) and signal recognition particle 54 kDa protein (SRP54), the stress response: ubiquitin-conjugating enzyme E2 J1 (UBE2J1), myeloid-derived growth factor (MYDGF), cysteine-rich with EGF-like domain protein 2 (CRELD2) and hypoxia upregulated protein 1/oxygen-regulated protein 150 kDa (HYOU1), immune activation: inter-alpha-trypsin inhibitor heavy chain H2 (ITIH2), afamin (AFM), plasma kallikrein (KLKB1), complement factor B (CFB) and carbonic anhydrase 3 (CA3), and the regulation of cell death: Band 3 anion transport protein/solute carrier family 4 member 1 (SLC4A1), ankyrin-1 (ANK1) and spectrin beta chain, erythrocytic (SPTB) (Figure 4B). In contrast, in SCC, processes associated with nucleotide metabolism, DNA damage repair, and the cell cycle: DNA replication licensing factor MCM2, 3,5,6,7 (MCMs-2,3,5,6,7) and proliferating cell nuclear antigen (PCNA), methylation: serine/arginine-rich splicing factor 11 (SRSF11), mitochondrial mRNA pseudouridine synthase RPUSD3 (RPUSD3), ATP-binding proteins: peroxisomal biogenesis factor 19 (PEX19), and drug metabolism: thymidine phosphorylase (TYMP), S100 calcium binding protein A2 (S100A2) and plakophilin-1 (PKP1), among others, were enriched (Figure 4C).

Among the 421 DEPs shared between HPV16+ LSIL and HPV16+ SCC and associated with overexpression, the enriched processes included gene expression: RNA-binding protein 42 (RBM42), fibrinogen alpha chain (FGA), RNA-binding motif protein, X chromosome (RBMX), antioxidant activity: hemoglobin subunit gamma-1 (HBG1), hemoglobin subunit delta (HBD), peroxiredoxin-1,2,5 (PRDX1/2/5) and glutathione S-transferase P (GSTP1), regulation of apoptosis: B-cell lymphoma/leukemia 10 protein (BCL10), keratin, type I cytoskeletal 18 (KRT18), 40S ribosomal protein S3a/Small ribosomal subunit protein eS1 (RPS3A) and 40S ribosomal protein S6/small ribosomal subunit protein uS6 (RPS6), and RNA binding: histone H1.2 (H1-2), high mobility group protein B2 (HMGB2), endothelial differentiation-related factor 1 (EDF1) and calcium-regulated heat-stable protein 1 (CARHSP1). This shared overexpressed set reflects a coordinated activation of transcriptional, post-transcriptional, antioxidant, and stress response programs common to both stages (Supplementary Figure S2).

Together, these results suggest a stage-specific shift during progression: LSIL is characterized by downregulation of reproductive tissue, membrane, and cell junction functions and upregulation of stress–response, protein–metabolism, and immune–activation processes, whereas SCC shows downregulation of adaptive immunity and energy metabolism and upregulation of DNA damage response, methylation, the cell cycle, and nucleotide and drug metabolism.

### 3.4. DEPs Associated with HPV16 Oncoproteins (E5, E6, and E7) and the Replicative Protein E1

**Figure 5:**
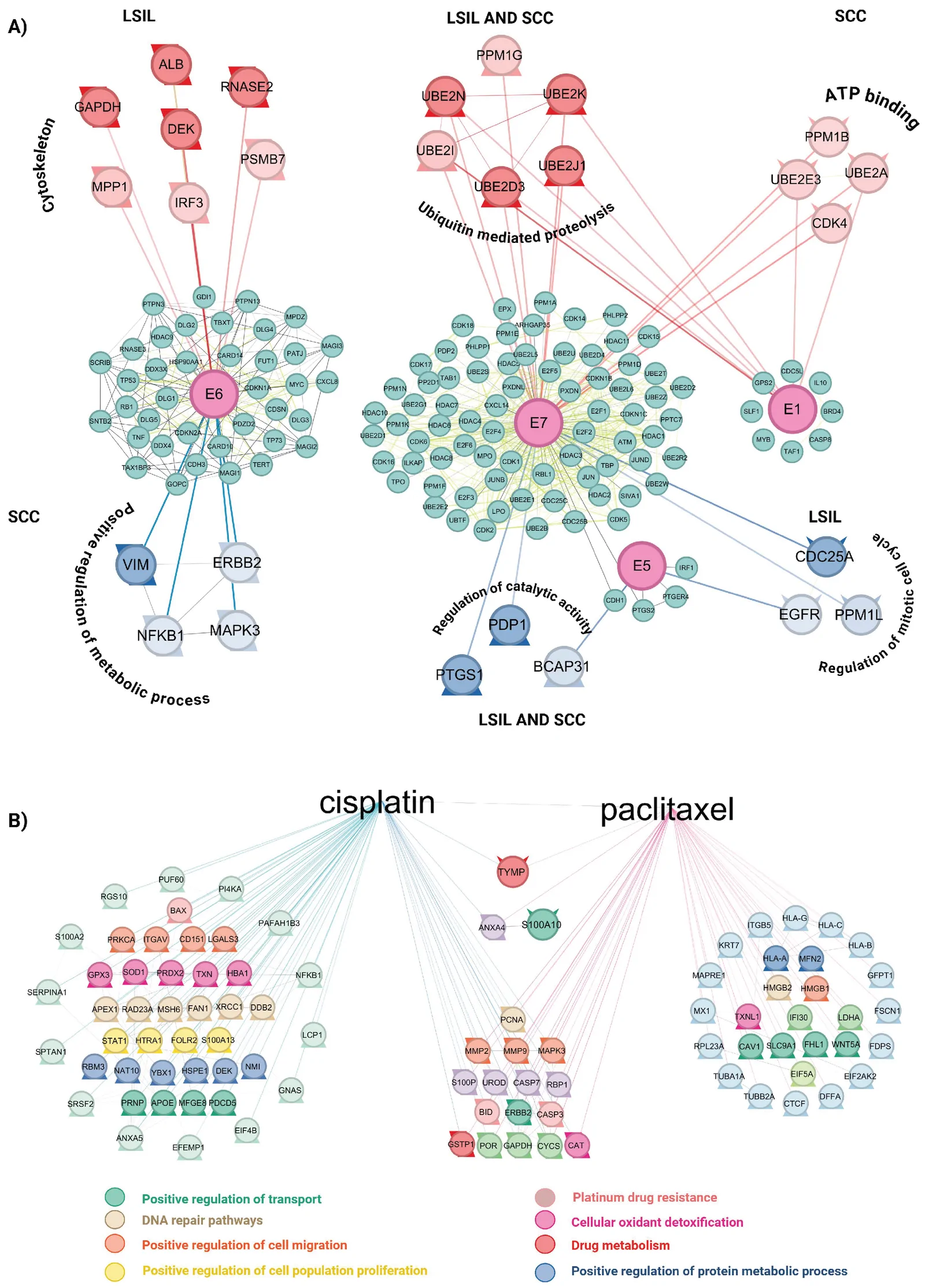
Interactome of DEPs in LSIL HPV16+ and SCC HPV16+ associated with viral proteins E1, E5, E6, and E7 and first-line chemotherapeutics. (**A**) Interactome of the HPV16 viral proteins E1, E5, E6, and E7 (pink color), reference *Homo sapiens* host proteins (green color) and interaction of DEPs in LSIL HPV16+ and SCC HPV16+. (**B**) Cisplatin/paclitaxel and DEPs interactomes from LSIL HPV16+ and SCC HPV16+.

To explore the likely interactions of the over- and underexpressed DEPs with the HPV16 viral proteins, a protein–protein interaction network was built in Cytoscape using human interactions retrieved from STRING (via stringApp); the HPV16 proteins (E1, E5, E6, E7) were incorporated from the Viruses STRING and were linked to their host targets. Functional enrichment of the network nodes was then performed against Gene Ontology “Biological Process” terms (adjusted *p*-value (FDR) < 0.05). A second interactome was constructed in the same way between the DEPs and the two first-line chemotherapeutic agents used in cervical cancer, cisplatin and paclitaxel, using drug–protein interactions from the STITCH database. The results revealed that proteins overexpressed in LSILs interact primarily with viral E6 oncoproteins, proteasome 20S subunit beta 7 (PSMB7), albumin (ALB), DEK proto-oncogene (DEK), membrane palmitoylated protein 1 (MPP1), interferon regulatory factor 3 (IRF3), ribonuclease A family member 2 (RNASE2) and GAPDH and are associated mainly with the proteolysis-related ubiquitination process. The underexpressed proteins M-phase inducer phosphatase 1/Cell division cycle 25A (CDC25A), protein phosphatase, Mg^2+^/Mn^2+^dependent 1L (PPM1L), and epidermal growth factor receptor (EGFR) were primarily associated with E7. The bioinformatic analysis of protein–protein interactions revealed a predicted association between E5 and cadherin-1 (CDH1), prostaglandin-endoperoxide synthase 2 (PTGS2), prostaglandin E2 receptor EP4 subtype (PTGER4), and interferon regulatory factor 1 (IRF1), as determined by the STRING database. When comparing this network with the set of DEPs identified in this study, it was observed that these same four proteins and epidermal growth factor receptor (EGFR) and B-cell receptor-associated protein 31 (BCAP31) have an additional, theoretical association with E5, suggesting a possible functional convergence between this oncoprotein and the differential proteomic profile observed in the patients analyzed (Figure 5A).

In the case of SCC, the overexpressed proteins ubiquitin conjugating enzyme E2 E3 (UBE2E3), ubiquitin conjugating enzyme E2 A (UBE2A), cyclin-dependent kinase 4 (CDK4), and protein phosphatase, Mg^2+^/Mn^2+^-dependent 1B (PPM1B) were mainly associated with E7 and E1. In contrast, the underexpressed vimentin (VIM), ERBB2, nuclear factor kappa B subunit 1 (NFKB1), and mitogen-activated protein kinase 3 (MAPK3) were associated with E6 and processes of regulation of catalytic activity and regulation of the cellular cycle. Interestingly, within the group of proteins shared between LSIL and SCC, an association was observed between E7 and the overexpressed proteins ubiquitin conjugating enzyme E2 I (UBE2I), protein phosphatase, Mg^2+^/Mn^2+^ dependent 1G (PPM1G), ubiquitin conjugating enzyme E2 J1 (UBE2J1), ubiquitin conjugating enzyme E2 K (UBE2K), ubiquitin conjugating enzyme E2 D3 (UBE2D3), and ubiquitin conjugating enzyme E2N (UBE2N), as well as the underexpressed pyruvate dehydrogenase phosphatase catalytic subunit 1 (PDP1), prostaglandin-endoperoxide synthase 1 (PTGS1), and B-cell receptor-associated protein 31 (BCAP31), which are associated with pathways of cancer progression and tubulin-related complexes. Interestingly, only proteins overexpressed in LSILs interacted with the E6 oncoprotein, whereas processes related to low-grade lesions and cervical cancer were associated with E7.

Additionally, to explore the possible relationships between the differentially expressed proteins (DEPs) and the first-line chemotherapeutic agents used in cervical cancer, cisplatin and paclitaxel, a drug–protein interaction network was constructed in Cytoscape (v. 3.10.2). The DEPs were integrated using stringApp, and drug–protein associations were retrieved from STITCH. The resulting network comprised 91 nodes and 107 edges (Figure 5B). Several proteins were predicted to interact with cisplatin. These included BCL2-associated X, apoptosis regulator (BAX), protein kinase C alpha (PRKCA), integrin subunit alpha V (ITGAV), CD151 molecule (CD151), and galectin 3 (LGALS3), which participate in the positive regulation of cell migration; glutathione peroxidase 3 (GPX3), superoxide dismutase 1 (SOD1), peroxiredoxin 2 (PRDX2), thioredoxin (TXN), and hemoglobin subunit alpha 1 (HBA1), which are associated with cellular detoxification; and signal transducer and activator of transcription 1 (STAT1), HtrA serine peptidase 1 (HTRA1), folate receptor beta (FOLR2), and S100 calcium binding protein A13 (S100A13), which are involved in the positive regulation of proliferation. For paclitaxel, the predicted interactors included high mobility group box 1 and 2 (HMGB1 and HMGB2), which participate in DNA repair, together with major histocompatibility complex class I A (HLA-A) and mitofusin 2 (MFN2). The analysis also identified proteins predicted to interact with both drugs. Among these, matrix metallopeptidase 2 (MMP2), MMP9, and mitogen-activated protein kinase 3 (MAPK3) participate in the positive regulation of cell migration, whereas glutathione S-transferase pi 1 (GSTP1) is associated with drug metabolism. S100A10 and TYMP were likewise predicted to interact with both cisplatin and paclitaxel: TYMP is involved in drug metabolism, whereas S100A10, which interacts indirectly through annexin A4 (ANXA4), plays an essential role in the positive regulation of molecular transport (Figure 5B).

These interactions suggest that the E6- and E7-associated DEPs converge on ubiquitination/proteolysis and cell cycle control, consistent with their proposed roles in progression.

### 3.5. Expression of S100A10 and TYMP Proteins in Patients with LSILs, HSILs, and SCC

**Figure 6:**
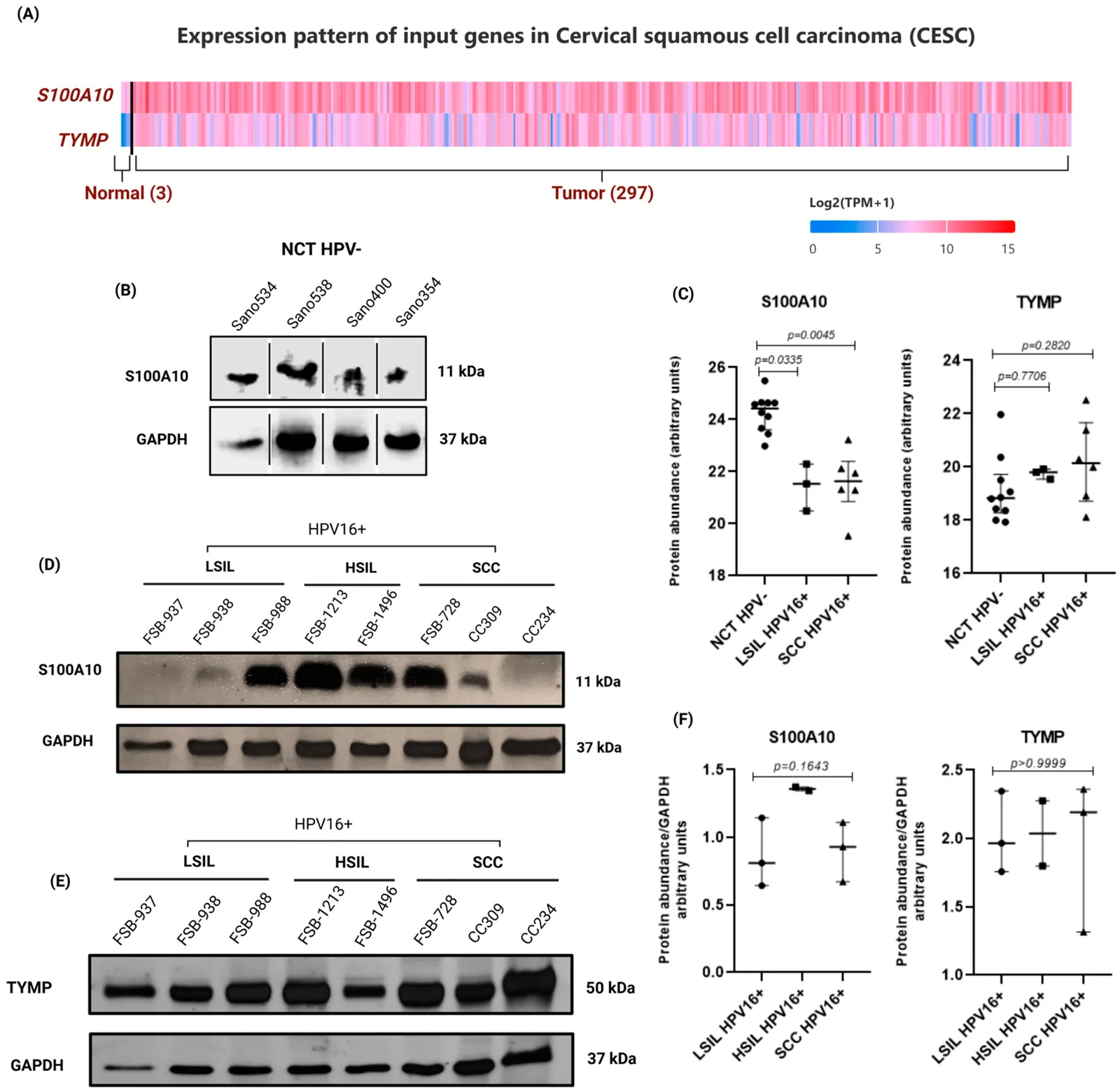
Analysis of S100A10 and TYMP in biopsies from patients with premalignant lesions and SCC. (**A**) Transcript-level expression of *S100A10* and *TYMP* in the TCGA-CESC dataset; samples are ordered as normal cervical tissue (left) and tumor (right), separated by a vertical divider, with the color scale indicating log_2_ (TPM + 1) (range 0–15). (**B**) Western blot of S100A10 in NCT HPV− biopsies. (**C**) Relative protein abundance from the proteomic (HPLC-MS/MS) analysis of S100A10 and TYMP in the NCT HPV− (n =10), LSIL HPV16+ (n =3) and SCC HPV16+ (n =6) groups. (**D**) Western blot of S100A10 in premalignant lesions and SCC. (**E**) Western blot of TYMP in premalignant lesions and SCC. (**F**) Relative protein expression by Western blot densitometry, normalized to GAPDH, for S100A10 and TYMP in the LSIL (n =3), HSIL (n =2), and SCC (n =3) groups. Each point represents one patient specimen; data are presented as medians with interquartile ranges, and differences among groups were assessed by the Kruskal–Wallis test followed by Dunn’s multiple-comparisons test. In the proteomic analysis (**C**), S100A10 was significantly lower in LSIL (*p* = 0.0335) and SCC (*p* = 0.0045) than in NCT HPV−, whereas TYMP did not differ significantly (LSIL *p* = 0.7706; SCC *p* = 0.2820). By Western blot densitometry (**F**), no statistically significant differences were observed (S100A10, *p* = 0.1643; TYMP, *p* > 0.9999).

Once the enriched processes associated with LSIL HPV16+ and SCC HPV16+ were established, and both shared and exclusive proteins and their interactions with viral oncoproteins were determined, two proteins, S100A10 and TYMP, were selected for further analysis. They were prioritized because they combined several features of interest: both were differentially expressed in our dataset with opposite, stage-relevant behavior—S100A10 was subexpressed in the LSIL compartment and TYMP was exclusively overexpressed in SCC; both appeared in the cisplatin/paclitaxel drug interactome (Figure 5B), linking them to treatment response; neither was a direct interactor of the viral oncoproteins (Figure 5A), positioning them as possible host-intrinsic candidates rather than direct viral effectors. To examine them, S100A10 and TYMP were analyzed at the transcript level in the TCGA-CESC dataset (Figure 6A), in the per-patient proteomic abundance data (Figure 6C), and by Western blot in normal cervical tissue (Figure 6B) and in premalignant and malignant lesions (Figure 6D–F). The mass spectrometry analysis was performed per patient, yielding the relative abundance of each protein for every specimen. S100A10 was significantly lower in LSIL HPV16+ (*p* = 0.0335) and SCC HPV16+ (*p* = 0.0045) than in NCT HPV−, consistent with its subexpression, whereas TYMP did not differ significantly between NCT HPV− and either LSIL (*p* = 0.7706) or SCC (*p* = 0.2820) (Kruskal–Wallis with Dunn’s test; Figure 6C). By Western blot, neither S100A10 nor TYMP differed significantly among the LSIL, HSIL, and SCC groups (Kruskal–Wallis *p* = 0.1643 and *p* > 0.9999, respectively; Dunn’s test, all pairwise comparisons non-significant; Figure 6F) (Supplementary Table S2).

At the proteomic level, S100A10 thus showed a significant, stage-associated decrease relative to normal tissue, whereas TYMP—identified as exclusive to SCC in the differential expression analysis—was also detectable in premalignant lesions but without a statistically significant difference between groups. Notably, neither protein was a direct interactor of the viral oncoproteins (Figure 5A), which positioned them as possible host-intrinsic candidates rather than direct viral effectors. Given the small sample size and the non-significant Western blot results, S100A10 and TYMP are presented as preliminary, hypothesis-generating candidates whose potential role in the progression of cervical lesions requires confirmation in larger, independent cohorts.

## 4. Discussion

The study of cervical cancer using a proteomic approach has enabled the identification of proteins with clinical relevance as potential biomarkers for diagnosis, cancer stratification, and treatment response, as well as for understanding the molecular mechanisms underlying the disease [25]. This study included samples from patients diagnosed with LSIL HPV16+ and SCC HPV16+, and both groups were compared with NCT HPV16- using HPLC-MS/MS with DIA, which enables reproducible protein identification and quantification with a relatively large dynamic range and high proteome coverage [26].

From the proteomic profiles obtained, shared and exclusive proteins were detected in premalignant lesions and CC. However, there are numerous proteomic studies of cervical cancer in various populations; proteomic studies in Latin America are limited [27], especially in Mexico [7,18]. The results obtained in this work allowed the identification of 1607 differentially expressed proteins: 693 were underexpressed, and 914 were overexpressed. There is little evidence from proteomic studies of LSILs; a survey of biopsies with cervical intraepithelial neoplasia grades 2 and 3 (CIN 2-3) lesions identified 165 proteins, of which zinc finger protein 441 (ZNF441) and phospholipase D6/mitochondrial cardiolipin hydrolase (PLD6) were associated with predicting CIN2 regression to CIN3 [28].

In a quantitative proteomics study using tandem mass tags (TMTs), 351 differentially expressed proteins (247 overexpressed and 104 underexpressed) were identified in SCC tissue compared with normal cervical tissue; these proteins were associated with apoptosis, immune response, and protein binding [29]. In a semiquantitative label-free proteomics study and DIA comparing SCC tissues and normal tissues, 562 differentially expressed proteins (340 overexpressed and 222 underexpressed) were identified, including proteins involved in metabolic pathways, the spliceosome, and cytoskeleton regulation [30].

In this study, we identified 242 proteins that were underexpressed exclusively in LSIL samples and associated with processes such as cell adhesion, membrane proteins, and the extracellular matrix integrin subunit alpha 3 (ITGA3), ITGA7, desmin (DES), collagen type XI alpha 1 chain (COL11A1), collagen type V alpha 3 chain (COL5A3), collagen type XXI alpha 1 chain (COL21A1), collagen type XXII alpha 1 chain (COL22A1), collagen type XXVIII alpha 1 chain (COL28A1), and collagen type IV alpha 6 chain (COL4A6). In this context, it has been reported that cervical epithelial cells can lose their morphology during mesenchymal-epithelial transition, which favors the progression of cervical lesions to SCC [31]. Proteins overexpressed in LSILs were related to processes such as immune response: complement C2, C3 and C5 (C2, C3, and C5); protein metabolism: ribosomal protein S27-like (RPS27L); signal recognition: particle 54 (SRP54), and ribosomal protein S26 (RPS26); stress response: (PRDX4), heat shock protein 90 beta family member 1 (HSP90B1), and UBE2J1; and cell death regulation: RAB29, member RAS oncogene family (RAB29), solute carrier family 4 member 1 (SLC4A1), and ATPase Na+/K+ transporting subunit beta 1 (ATP1B1).

Persistent HPV infection has been reported to reduce the expression of major histocompatibility complex (MHC) class I molecules in infected cells, thus preventing recognition by cytotoxic T cells [32]. In HPV16+ SCC, we observed similar enrichment patterns of subexpressed proteins, which were associated mainly with the immune response, including the presentation of MHC class II antigens (HLA-DRB1, HLA-DRB5, HLA-DRA, HLA-DPA1, and HLA-DQB1).

The proteins identified as overexpressed in HPV16+ SCC and associated with the cell cycle, such as minichromosome maintenance complex component 2, 3, 5, 6, and 7 (MCM2, MCM3, MCM5, MCM6, and MCM7), have been previously described [33]; however, MCM5 was also identified in this study. These proteins are essential for cell cycle proliferation and regulation. Similarly, proteins involved in metabolism, including BRCA2 and CDKN1A interacting protein (BCCIP), DEAD-box helicase 47 (DDX47), NSA2 ribosome biogenesis factor (NSA2), DNA fragmentation factor subunit alpha (DFFA), NME/NM23 nucleoside diphosphate kinase 1 (NME1), and G1 to S phase transition 2 (GSPT2), which participate in metabolic reprogramming, a hallmark of cervical cancer that supports the survival and development needs of patients with malignant tumors, were identified [34]. In cervical cancer, HPV infection also interferes with the deregulation of metabolic processes such as glycolysis [35], since lactate metabolism promotes the immunosuppressive polarization of macrophages [36]; lipid metabolism, which is related to malignant progression in cervical cancer [37] and glutamine metabolism, as it provides cancer cells with essential nutrients such as proteins, nucleic acids, and lipids for their constant proliferation [38].

On the other hand, when the interaction analysis of exclusive and shared proteins in LSIL HPV16+ and SCC HPV16+, both underexpressed and overexpressed with HPV16 oncoproteins, was performed using the interactome “HPV16–host protein interaction network” in Cytoscape, proteins with direct physical interactions between the oncoproteins and binding to PSD-95/Disks Large/Zonula Occludens-1 domain (PDZ) domains were observed. As in the case of the membrane-associated guanylate kinase, WW and PDZ domain-containing 1 (MAGI-1) protein, which interacts with E6 [39], recent evidence has shown that MAGI-1 expression can differentiate cervical cells with normal morphology from HPV16-infected cells in the integrated state [40]. In this work, the proteins overexpressed in both LSIL and SCC, UBE2K, PPM1G, UBE2I, UBE2D3, and UBE2N, and the underexpressed proteins PDP1, PTGS1, and BCAP31 interacted with the E7 oncoprotein. A previous study revealed that the E7 oncoprotein expression may serve as a predictive biomarker for progression from LSILs to HSILs and SCC [41]. It has also been reported that host proteins, such as UBE2I, may be overexpressed in LSILs and be associated with disease progression [42]. In contrast, overexpression of UBE2D3 induces evasion of antigen presentation via interferon-gamma (IFN-γ) [43], in addition to regulating protein ubiquitination, as UBE2D3 regulates the levels of the tumor suppressor protein p53 (p53) by regulating its proteasomal degradation [44]. The PPM1G protein, which is overexpressed in LSIL HPV16+ and SCC HPV16+, is associated with tumor progression through its direct interaction with P53, as observed in hepatocarcinoma cells, where dephosphorylation of ubiquitin carboxyl-terminal hydrolase 7 (USP7) can lead to P53 degradation [45]. UBE2N was found to be overexpressed in cervical carcinoma and is associated with decreased survival [46], findings in agreement with those for the UBE2N protein. However, its role in SCC has not been fully elucidated, although it is involved in DNA repair [47] and plays a fundamental role in the development of cisplatin chemoresistance and metastasis [48].

E5 is currently considered a potential viral oncoprotein with multiple mechanisms of action on the host cell. Through co-immunoprecipitation assays, it has been demonstrated that the E5 variants of HPV16 and HPV31 physically associate with BCAP31 (BAP31), a chaperone involved in the transport of MHC-I molecules in the endoplasmic reticulum; interference targeting BCAP31 reduces the proliferative and differentiation capacity of infected keratinocytes, positioning this interaction as a key mechanism in the maintenance of the proliferative phenotype [49]. Additionally, E5 amplifies EGFR signaling, indirectly promoting the creation of a microenvironment conducive to viral replication [50]. Recent studies have shown that the interaction of E5 with proteins such as BCAP31 promotes its relocalization to the endoplasmic reticulum, helping to sustain a state of active cell proliferation [51]. Beyond its role in proliferation, E5 exerts a significant influence on the modulation of the innate immune response: it has been reported to suppress the production of IFN-κ and IFN-β through sustained activation of the EGFR/MAPK axis, which reduces IRF1 transcriptional activity by limiting its access to the IFN-κ promoter [52]. Taken together, these findings support the bioinformatic association observed between E5 and IRF1 (Figure 5A), and suggest that the convergence with CDH1, PTGS2, and PTGER4 may reflect a shared functional network involving cell proliferation, immune evasion, and microenvironment remodeling.

In the interaction analysis of DEPs with HPV16 proteins in SCC, UBE2E3 and UBE2A were found to interact with the E1 protein. Recently, it has been suggested that the E1 protein, while essential for viral replication, also interacts with cellular components that affect cancer progression [53]. Recent reports indicate that E1 plays a role in regulating epigenetic modulation in cells and controlling replication mechanisms [54]. A recent study described the association of E1 proteins with immune responses, including TLR and interferon signaling, and revealed that HPV16 infection increases immune tolerance, increasing the susceptibility of the microenvironment to new infections and disease progression [55]. E1 has recently been reported to regulate the epigenetic modulation of cells, which are involved in the suppression of interferon beta 1 (IFNβ1) and interferon lambda 1 (IFNλ1) expression via the NF-κB and Janus kinase-signal transducer and activator of transcription (JAK-STAT) pathways and in interfering with disease progression [55,56].

The enrichment of cell adhesion, extracellular matrix, and immune activation processes in LSIL and their replacement by proliferative, metabolic, cell cycle, and DNA damage programs in SCC is consistent with an early, reversible host response (barrier remodeling and immune engagement) that is progressively lost or reprogrammed as lesions acquire a malignant, proliferation-driven phenotype. This pattern suggests a possible molecular transition during progression and may be relevant for the definition of stage-specific markers. Processes such as cell cycle/MCM activity, metabolic reprogramming, and MHC-related immune evasion are consistent with previous reports [36,38,57], whereas other observations, such as the LSIL-exclusive adhesion/ECM and reproductive-tissue signature, and the identification of MCM5, have not, to our knowledge, been reported in this context.

To address how the viral proteins may act on the altered processes along progression, we examined a functional interactome built between the DEPs and the HPV16 proteins (E1, E5, E6, and E7; Figure 5A). This network places the host DEPs in the context of their predicted viral interactions and is particularly informative for the shared DEPs differentially expressed in both LSIL and SCC. The overexpressed ubiquitin conjugating enzymes and phosphatase shared by both stages (UBE2I, UBE2K, UBE2N, UBE2D3, UBE2J1, PPM1G) and the shared downregulated PDP1, PTGS1, and BCAP31 were associated mainly with E7 (Figure 5A). Because these proteins are altered from the low-grade stage onward and remain altered in cancer, they suggest that E7 engages the ubiquitin–proteasome and cell cycle machinery continuously throughout progression, not only in established carcinoma. In parallel, the LSIL-restricted E6 interactions (e.g., with PSMB7, ALB, DEK, IRF3) and the SCC-restricted, E1-associated UBE2E3/UBE2A point to stage-specific contributions: an early E6-driven remodeling of proteolysis and immune signaling that gives way to E7- and E1-associated proliferative and replication programs in SCC. These links are discussed considering the established roles of E6 in p53 degradation and PDZ target disruption, E7 in Rb/E2F activation, and the reported functions of UBE2D3, UBE2N, PPM1G, and UBE2I in HPV−associated disease [39,40,41,42,43,44,45,46,47,48].

The first-line chemotherapeutic drugs used for the treatment of cervical cancer are cisplatin and paclitaxel [58]. It has been reported that cisplatin causes DNA damage, whereas paclitaxel interferes with the cell cycle by blocking mitosis [59]. Dysregulated cellular processes associated with chemoresistance to both drugs, such as DNA damage repair [60], drug transport [61], cell death regulation [62], and epigenetic reprogramming [63], have been described. Interaction analysis revealed that proteins such as X-ray repair cross-complementing 1 (XRCC1), mutS homolog 6 (MSH6), FANCD2, and FANCI associated nuclease 1 (FAN1), proliferating cell nuclear antigen (PCNA), and damage-specific DNA binding protein 2 (DDB2) are associated with DNA repair, as are GSTP1 and TYMP, which are related to cellular detoxification. GSTP1 has been reported to be associated with cisplatin resistance through the downregulation of reactive oxygen species (ROS) levels [64], whereas TYMP inhibition increases cisplatin sensitivity in bladder cancer [65]. The mechanisms of paclitaxel resistance are associated mainly with drug flux and efflux [66] and cellular metabolism [67]. In this study, the proteins HLA-A and MFN2 were identified as regulators of metabolic processes. Interestingly, proteins associated with both cisplatin and paclitaxel, such as MMP2, MMP9, and MAPK3, which are related to the process of cell migration, and the transport proteins ERBB2 and S100A10, which are directly associated with ANXA4 (promoting cell proliferation and regulating the cell cycle [68] and whose overexpression increases resistance to cisplatin in ovarian cancer [69]) were identified.

The analysis was intended to identify, among our progression-associated DEPs, candidate host proteins that intersect the known response pathways of these first-line drugs (DNA-damage repair, detoxification, drug metabolism, and transport). This includes both proteins shared across LSIL and SCC and proteins exclusive to SCC, several of which, such as GSTP1, TYMP, S100A10/ANXA4, MMP2/MMP9, and MAPK3, had not previously been linked to cisplatin/paclitaxel response in HPV16+ cervical disease in this combined manner. It should be emphasized, however, that these relationships derive exclusively from an in silico interaction network built from curated and text-mined associations (STITCH); therefore, they represent computationally predicted associations rather than experimentally established links. Experimental approaches, such as knockdown or overexpression of S100A10 or TYMP followed by cytotoxicity and chemoresistance assays, will be required before any role of these proteins in the response to cisplatin or paclitaxel can be established. Accordingly, the significance of these findings is hypothesis-generating: the identified proteins are nominated as candidates that may contribute to intrinsic chemoresistance and as potential targets for future therapeutic strategies. Because drug resistance was not experimentally tested in the present study, these associations should be regarded as provisional and warrant functional and clinical validation.

Among the DEPs, S100A10 and TYMP were selected for further analysis because they combined several features of interest: they showed opposite, stage-relevant behavior; S100A10 was subexpressed in the LSIL, whereas TYMP was exclusively overexpressed in SCC. Both appeared in the cisplatin/paclitaxel drug interactome (Figure 5B), linking them to treatment response. Notably, neither was a direct interactor of the viral oncoproteins (Figure 5A), which positioned them as possible host-intrinsic markers of progression rather than direct viral effectors. Their overexpression was independently supported at the transcript level in the TCGA-CESC dataset. However, the Western blot analysis did not reach statistical significance; S100A10 and TYMP are presented here as preliminary candidates that require validation in larger, independent cohorts and functional studies. 

S100A10 belongs to the S100 protein family, has a molecular weight of 11 kDa, and can modulate, alone or in combination with annexin A2, ion channels and receptors, regulating their localization and function in cells [70]. S100A10 is a plasminogen receptor that promotes an invasive phenotype in cancer cells [71]. Its positive expression has been evaluated in patients with SCC, and its overexpression has been associated with poor prognosis [72]. S100A10 also plays a protumorigenic role, regulating processes such as proliferation, cell adhesion, migration, invasion, metastasis, and treatment resistance in various malignancies [73,74]. In colorectal cancer, S100A10 overexpression has been associated with oxaliplatin resistance [75].

The TYMP protein encodes thymidine phosphorylase (TP), an essential enzyme in pyrimidine nucleoside metabolism [76], has a molecular weight of 55 kDa, and is involved in tumor progression through its roles in proliferation, angiogenesis, and response to treatment [77]. TYMP is known to be overexpressed in cancer under cellular stress conditions such as hypoxia [78], acidosis [79], chemotherapy [80], and radiotherapy [81]. TYMP can also facilitate DNA repair mechanisms, including mismatch repair (MMR) [82] and homologous recombination repair (HR), which promote genomic stability, pluripotency, and chemotherapeutic resistance [83]. Increased TYMP expression leads to increased interleukin-10 (IL-10) secretion, which suppresses the immune response [84]. The use of TYMP-targeted inhibitors such as trifluridine and tipiracil (TAS-102) has been reported to induce tumor regression in preclinical gastrointestinal tumor models [85].

TYMP has been reported to be overexpressed in various cancers, including breast cancer [86], gastric cancer [87], renal cancer [88], and colorectal cancer [89]. In patients with cervical cancer, TYMP expression has been reported to be upregulated in patients undergoing chemotherapy or radiotherapy [79]. In this study, TYMP was exclusively overexpressed in SCC in the differential expression analysis but showed no statistically significant differences by proteomic quantification or Western blot; thus, S100A10 and TYMP are proposed as preliminary, hypothesis-generating candidates requiring validation before being considered therapeutic targets in SCC.

Several limitations should be considered when interpreting these results. First, the study is exploratory and based on a small, imbalanced cohort, particularly in the discovery phase (NCT HPV − n = 10, LSIL HPV16 + n = 3, SCC HPV16 + n = 6); this limits statistical power, reduces the stability of the differential expression estimates, makes false discovery-rate control more sensitive to individual specimens, and constrains the generalizability of the identified protein signatures. The cervical biopsies are intrinsically heterogeneous, comprising variable proportions of epithelial, stromal, and immune cells; because the proteomic analysis was performed on bulk tissue, part of the between-group differences may reflect differences in cell-population composition rather than cell-intrinsic molecular alterations. The TCGA-CESC dataset is predominantly a cervical-cancer cohort and therefore provides independent transcript-level data but does not include premalignant stages and cannot validate the LSIL/HSIL→SCC transition. The protein-level validation was restricted to two candidates (S100A10 and TYMP) in a small set of specimens (LSIL n = 3, HSIL n = 2, SCC n = 3), and the Western blot showed no statistically significant differences among groups; therefore, the absence of significance cannot be taken as evidence of no effect. Finally, the viral–host (E1/E5/E6/E7) and drug–protein (cisplatin/paclitaxel) interaction networks are bioinformatic predictions derived from curated databases and were not experimentally tested; they are presented as putative, hypothesis-generating associations rather than demonstrated interactions.

## 5. Conclusions

This study provides one of the first DIA-MS proteomic maps of HPV16+ cervical lesion progression, from LSIL to invasive SCC, in an understudied southern Mexican population, and identifies a set of candidate host proteins (including S100A10 and TYMP) and predicted viral–host interactions. Its main contribution is to delineate the processes that distinguish low-grade lesions from cancer and to nominate host-intrinsic candidates of progression that are not direct effectors of the viral oncoproteins, which may help guide the search for progression biomarkers and therapeutic targets. These findings provide exploratory proteomic candidates associated with HPV16-positive cervical lesions and SCC status, which warrant validation in larger, independent cohorts. Given the exploratory nature of the cohort, these findings are hypothesis-generating and define clear future directions: validation in larger, balanced, independent cohorts that include HSIL in the discovery phase; functional and co-immunoprecipitation studies to confirm the predicted viral–host interactions; and drug response assays to test whether the chemotherapy-linked candidates contribute to intrinsic resistance to cisplatin and paclitaxel.

## Data Availability

The mass spectrometry proteomics data have been deposited to the ProteomeXchange Consortium (https://proteomecentral.proteomexchange.org, access date: 9 February 2026) ) via the iProX partner repository [90,91] with the dataset identifier PXD074215.

## Supplementary Materials

The following supporting information can be downloaded at: https://www.mdpi.com/article/10.3390/pathogens15080793/s1. Supplementary Figure S1. Bioinformatic analysis of shared underexpressed proteins between LSIL HPV16+ and SCC HPV16+ groups. Supplementary Figure S2. Overexpressed proteins common to LSIL and SCC groups. Supplementary Table S1. Clinicopathological characteristics of the specimens used in this study. Supplementary Table S2. Raw protein abundance data obtained by HPLC-MS/MS analysis. Supplementary Table S3. Densitometric quantification of target proteins normalized to total protein (Ponceau S loading control).

## Abbreviations

14-3-3ζ	14-3-3 protein zeta/delta
2D-DIGE	Two-Dimensional Difference Gel Electrophoresis
ACTA2	Actin Alpha 2, Smooth Muscle
AFM	Afamin
AGC	Automatic Gain Control
ALB	Albumin
ANK1	Ankyrin-1
ANXA4	Annexin A4
ANXA6	Annexin A6
ATP1B1	Atpase Na+/K+ Transporting Subunit Beta 1
BAX	BCL2 Associated X, Apoptosis Regulator
BCAP31	B Cell Receptor Associated Protein 31
BCCIP	BRCA2 And CDKN1A Interacting Protein
BCL10	B-cell lymphoma/leukemia 10 protein
C2/C3/C5	Complement C2, C3, and C5
CA3	Carbonic anhydrase 3
CARHSP1	Calcium-regulated heat-stable protein 1
CAV1	Caveolin-1
CC	Cervical Cancer
CD151	CD151 Molecule
CDC25A	M-Phase Inducer Phosphatase 1/Cell Division Cycle 25A
CDH1	Cadherin-1
CDK4	Cyclin Dependent Kinase 4
CFB	Complement factor B
CIN/CIN2/CIN3	Cervical Intraepithelial Neoplasia (Grades 2 And 3)
COL11A1	Collagen Type XI Alpha 1 Chain
COL21A1	Collagen Type XXI Alpha 1 Chain
COL22A1	Collagen Type XXII Alpha 1 Chain
COL28A1	Collagen Type XXVIII Alpha 1 Chain
COL4A6	Collagen Type IV Alpha 6 Chain
COL5A3	Collagen Type V Alpha 3 Chain
COL6A2	Collagen alpha-2(VI) chain
CRELD2	Cysteine-rich with EGF-like domain protein 2
DCN	Decorin
DDA	Data-Dependent Acquisition
DDB2	Damage Specific DNA Binding Protein 2
DDX47	DEAD-Box Helicase 47
DEK	DEK Proto-Oncogene
DEPs	Differentially Expressed Proteins
DES	Desmin
DFFA	DNA Fragmentation Factor Subunit Alpha
DIA	Data-Independent Acquisition
DIA-NN	Data-Independent Acquisition Neural Networks (Software)
DNA/RNA/mRNA	Deoxyribonucleic Acid/Ribonucleic Acid/Messenger RNA
DTT	Dithiothreitol
E1, E5, E6 and E7	Early Proteins 1,5,6 And 7
EDF1	Endothelial differentiation-related factor 1
EGFR	Epidermal Growth Factor Receptor
AGO2	Argonaute RISC Catalytic Component 2
ENO1	Enolase 1
EP300	E1A Binding Protein P300
ERBB2	Erb-B2 Receptor Tyrosine Kinase 2
FAM179B	TOG Array Regulator Of Axonemal Microtubules 1
FAN1	FANCD2 And FANCI Associated Nuclease 1
FDR	False Discovery Rate
FGA	Fibrinogen alpha chain
FOLR2	Folate Receptor Beta
FOSL2/FOSL2-K222	FOS-Like Antigen 2/Acetylation At Lysine 222
FOXM1-ABCC5	Forkhead Box M1—ATP-Binding Cassette Subfamily C Member 5 Axis
GAPDH	Glyceraldehyde-3-Phosphate Dehydrogenase
GLI2	GLI Family Zinc Finger 2
GNB1/GNB2/GNB4	Guanine nucleotide-binding protein G(I)/G(S)/G(T) subunit beta-1,2, and 4
GO	Gene Ontology
GPX3	Glutathione Peroxidase 3
GSPT2	G1 To S Phase Transition 2
GSTP1	Glutathione S-Transferase Pi 1
H1-2	Histone H1.2
HBA1	Hemoglobin Subunit Alpha 1
HBD	Hemoglobin subunit delta
HBG1	Hemoglobin subunit gamma-1
HLA-A/HLA-DRA/HLA-DRB1/HLA-DRB5/HLA-DPA1/HLA-DQB1	Human Leukocyte Antigens (Classes I And II)
HMGB1	High Mobility Group Box 1
HMGB2	High Mobility Group Box 2
HPLC-MS/MS	High-Performance Liquid Chromatography-Tandem Mass Spectrometry
HR	Homologous Recombination
HR-HPV	High-Risk Human Papillomavirus
HRP	Horseradish Peroxidase
HSP90B1	Heat Shock Protein 90 Beta Family Member 1
HTRA1	Htra Serine Peptidase 1
HYOU1	Hypoxia up-regulated protein 1/Oxygen-regulated protein 150 kDa
IFNβ1	Interferon Beta 1
IFN-γ,	Interferon Gamma
IFNλ1	Interferon Lambda 1
IL-10	Interleukin-10
INNO-LIPA	Innogenetics Line Probe Assay (HPV Genotyping)
IRF1	Interferon regulatory factor 1
IRF3	Interferon Regulatory Factor 3
RPUSD3	Mitochondrial mRNA pseudouridine synthase RPUSD3
ITGA3	Integrin Subunit Alpha 3, 7, and V
ITGB5	Integrin subunit beta 5
ITIH2	Inter-alpha-trypsin inhibitor heavy chain H2
JAK-STAT	Janus Kinase-Signal Transducer And Activator Of Transcription Pathway
KLKB1	Plasma kallikrein
KRT18	Keratin, type I cytoskeletal 18
KRT5	Keratin 5
LAMB2	Laminin subunit beta 2
LC-MS/MS	Liquid Chromatography-Tandem Mass Spectrometry
LGALS3	Galectin 3
LRP1B	LDL Receptor Related Protein 1B
LSIL	Low-Grade Intraepithelial Lesion
LUM	Lumican
MACF1	Microtubule Actin Crosslinking Factor 1
MAGI-1	Membrane Associated Guanylate Kinase, WW And PDZ Domain Containing 1
MALDI-TOF-MS	Matrix-Assisted Laser Desorption/Ionization Time-Of-Flight Mass Spectrometry
MAPK3	Mitogen-Activated Protein Kinase 3
MBR	Match Between Runs
MCM2/3/5/6/7/10	Minichromosome Maintenance Complex Components 2,3,5,6,7, and 10
MFN2	Mitofusin 2
MHC	Major Histocompatibility Complex
MMP2/MMP9	Matrix Metalloproteinase 2/9
MMR	Mismatch Repair
MPP1	Membrane Palmitoylated Protein 1
MS1/MS2	Mass Spectrometry Stage 1/Stage 2
MS-DAP	Mass Spectrometry Downstream Analysis Pipeline
MSH6	Muts Homolog 6
MUC12	Mucin 12, Cell Surface Associated
MUC4	Mucin 4, Cell Surface Associated
MYC	MYC Proto-Oncogene
MYDGF	Myeloid-derived growth factor
NCE	Normalized Collision Energy
NCT HPV−	HPV−Negative Normal Cervical Tissue
NES	Nestin
NFKB1	Nuclear Factor Kappa B Subunit 1
NME1	NME/NM23 Nucleoside Diphosphate Kinase 1
NR4A2	Nuclear Receptor Subfamily 4 Group A Member 2
NSA2	NSA2 Ribosome Biogenesis Factor
OBSCN	Obscurin, Cytoskeletal Calmodulin And Titin-Interacting Rhogef
OGN	Osteoglycin
p53	Tumor Protein P53
PCA	Principal Component Analysis
PCNA	Proliferating Cell Nuclear Antigen
PDP1	Pyruvate Dehydrogenase Phosphatase Catalytic Subunit 1
PDZ	PSD-95/Discs Large/Zonula Occludens-1 Domain
PEX19	Peroxisomal biogenesis factor 19
PIK3CA	Phosphatidylinositol-4,5-Bisphosphate 3-Kinase Catalytic Subunit Alpha
PKHD1L1	Fibrocystin-L/Polycystic Kidney And Hepatic Disease 1-Like Protein 1
PKP1	Plakophilin-1
PLD6	Phospholipase D6/Mitochondrial Cardiolipin Hydrolase
PLEC	Plectin
PPM1B	Protein Phosphatase, Mg^2+^/Mn^2+^ Dependent 1B
PPM1G	Protein Phosphatase, Mg^2+^/Mn^2+^ Dependent 1G
PPM1L	Protein Phosphatase, Mg^2+^/Mn^2+^ Dependent 1L
PRDX1	Peroxiredoxin 1
PRDX2	Peroxiredoxin 2
PRDX4	Peroxiredoxin 4
PRDX5	Peroxiredoxin-5, mitochondrial
PRKCA	Protein Kinase C Alpha
PRKCB	Protein Kinase C Beta
PROX1	Prospero Homeobox 1
PSMB7	Proteasome 20S Subunit Beta 7
PTGER4	Prostaglandin E2 receptor EP4 subtype
PTGS1	Prostaglandin-Endoperoxide Synthase 1
PTGS2	Prostaglandin-endoperoxide synthase 2
PTMs	Post-Translational Modifications
RAB29	RAB29, Member RAS Oncogene Family
RBM42	RNA-binding protein 42
RBMX	RNA-binding motif protein, X chromosome/Heterogeneous nuclear ribonucleoprotein G
RNASE2	Ribonuclease A Family Member 2
ROS	Reactive Oxygen Species
RPS26	Ribosomal Protein S26
RPS27L	40S ribosomal protein S27-like
RPS3A	40S ribosomal protein S3a/Small ribosomal subunit protein eS1
RPS6	40S ribosomal protein S6/Small ribosomal subunit protein uS6
RT	Room Temperature
S100A10	S100 Calcium Binding Protein A10 (P11)
S100A13	S100 Calcium Binding Protein A13
S100A2	S100 Calcium Binding Protein A2
SCC	Squamous Cell Carcinoma
SDS	Sodium Dodecyl Sulfate
SEC11C	Signal peptidase complex catalytic subunit SEC11C
SERPINB4	Serpin Family B Member 4
SFN	Stratifin
SLC4A1	Solute Carrier Family 4 Member 1
SOD1	Superoxide Dismutase 1
SPTB	Spectrin beta chain, erythrocytic
SRP54	Signal Recognition Particle 54
SRSF11	Serine/arginine-rich splicing factor 11
STAT1	Signal Transducer And Activator Of Transcription 1
TAGLN	Transgelin
TAS-102	Trifluridine/Tipiracil Combination Drug
TBS/TBS-T	Tris-Buffered Saline/TBS With Tween-20
TCGA	The Cancer Genome Atlas
TCGA-CESC	TCGA Cervical Squamous Cell Carcinoma Dataset
TFA	Trifluoroacetic Acid
TMT	Tandem Mass Tags
TNIK	NCK Interacting Kinase
TNXB	Tenascin-X
TXN	Thioredoxin
TYMP	Thymidine Phosphorylase
UALCAN	University Of Alabama Cancer Database
UBE2A	Ubiquitin Conjugating Enzyme E2 A
UBE2D3	Ubiquitin Conjugating Enzyme E2 D3
UBE2E3	Ubiquitin Conjugating Enzyme E2 E3
UBE2I	Ubiquitin Conjugating Enzyme E2 I
UBE2J1	Ubiquitin Conjugating Enzyme E2 J1
UBE2K	Ubiquitin Conjugating Enzyme E2 K
UBE2N	Ubiquitin Conjugating Enzyme E2 N
UniProt	Universal Protein Resource
UPLC	Ultra-Performance Liquid Chromatography
USP7	Ubiquitin Carboxyl-Terminal Hydrolase 7
VIM	Vimentin
VSN	Variance Stabilizing Normalization
XRCC1	X-Ray Repair Cross Complementing 1
ZNF441	Zinc Finger Protein 441
